# Chronic Eosinophilic Leukemia Presenting as Cardiac Failure

**DOI:** 10.1155/2022/7841310

**Published:** 2022-12-16

**Authors:** Nii Boi-Doku Pepra-Ameyaw, William Kwasi Ghunney, Eugene Baafi Ampofo, Edeghonghon Olayemi

**Affiliations:** ^1^Department of Haematology, Korle Bu Teaching Hospital, Accra, Ghana; ^2^Ghana Institute of Clinical Genetics, Korle Bu, Accra, Ghana; ^3^National Cardiothoracic Centre, Korle Bu Teaching Hospital, Accra, Ghana; ^4^Department of Haematology, University of Ghana Medical School, Accra, Ghana

## Abstract

Chronic eosinophilic leukemia (CEL) is a rare chronic myeloproliferative disorder characterized by sustained eosinophilia. Although the incidence of CEL is uncertain, it can be clinically devastating as it has a propensity to affect several important organ systems. This is of particular significance in Sub-Saharan Africa where helminthic infections are a more prevalent cause of eosinophilia. To the best of our knowledge, we present the first reported case of CEL complicated by cardiac disease in a Ghanaian. He presented with a history of orthopnoea and dyspnoea on exertion, and examination revealed a pansystolic murmur over the mitral region and moderate splenomegaly. Good symptomatic control was achieved using hydroxyurea after which haematologic and cytogenetic remission was achieved after 12 weeks on a tyrosine kinase inhibitor. Physicians working in low resource environments should exclude clonality in patients presenting with eosinophilia and end-organ damage.

## 1. Introduction

Chronic eosinophilic leukemia (CEL) is a rare chronic myeloproliferative disorder characterized by an absolute eosinophil count greater than 1.5 × 10^9^ cells/L persisting for more than 6 months and the presence of myeloid cells that exhibit a clonal cytogenetic aberration [1]. It shares a clinicopathological overlap with idiopathic hypereosinophilic syndrome in which tissue damage is present without an associated identifiable cytogenetic aberration or blasts greater than 5% but less than 20% in the bone marrow [2]. In the United States, the age-adjusted incidence of hypereosinophilic syndrome is estimated to be approximately 0.036 per 100,000 person-years [3]. Eosinophilia with recurrent genetic abnormalities make up an even smaller subset of these cases.

The Revised 2016 World Health Organization Classification of Eosinophilic Disorders recognizes myeloid/lymphoid neoplasms with eosinophilia and rearrangement of PDGFRA, PDGFRB, or FGFR1, or with PCM-JAK2 as a distinct clinical entity [1]. The incidence of *FIP1L1-PDGFRA* in patients with idiopathic hypereosinophilia is approximately 10% [4]; this is of significant clinical consequence as this group of patients is uniquely sensitive to tyrosine kinase inhibitor therapy and is capable of achieving complete molecular remission [5]. *CHIC2* deletion is a surrogate for the *FIP1L1-PDGFRA* fusion.

## 2. Case Presentation

A 37-year-old man with no known history of hypertension, atherosclerosis, or heart disease presented with a year-long history of progressively worsening dyspnoea on exertion associated with orthopnoea and bipedal oedema. He had no known history of allergic disease or exposure to toxins. At presentation, his blood pressure was 90/67 mmHg. Further examination revealed a chronically ill looking adult male with raised jugular venous pressure and pitting bipedal oedema up to the level of the distal third of his tibia, a grossly enlarged spleen approximately 10 cm below the left costal margin, and ascites and bilateral inguinal lymphadenopathy. Auscultation revealed a pansystolic murmur over the mitral region.

**Figure 1:**
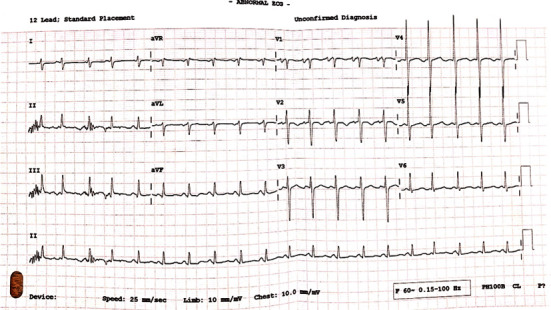
ECG at presentation showing sinus tachycardia and a right axis deviation. P wave duration of 120 ms with deep terminal portion > 1 mm (left atrial component) in V1 indicative of a dilated left atrium. The dilated left atrium is a result of an increased volume load from the severe mitral regurgitation.

**Figure 2:**
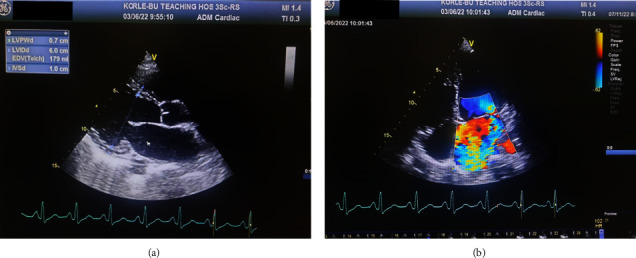
Echocardiogram 5 weeks post imatinib: (a) 2D parasternal long axis view showing dilated left ventricle, left atrium and thickened mitral valve leaflets. (b) 2D apical 4-chamber view showing severe mitral regurgitation on colour doppler.

Chest radiography displayed a cardiomegaly with cephalization of vessels. Sinus tachycardia and a right axis deviation were seen on the electrocardiogram (figure 1). An echocardiogram further revealed a thickened mitral valve with severe mitral regurgitation, mild tricuspid regurgitation, mild left atrial dilatation, mild pulmonary hypertension, and a pericardial effusion. Measured left ventricular ejection fraction was 63% (Figure 2). About 18 months prior, he had noticed insidious migratory pain that involved the shoulder, elbow, wrist, knee, and ankle joints, and this pain was severe enough to interfere with his activities of daily living with poor response to nonsteroidal anti-inflammatory drugs (NSAIDS).

**Figure 3:**
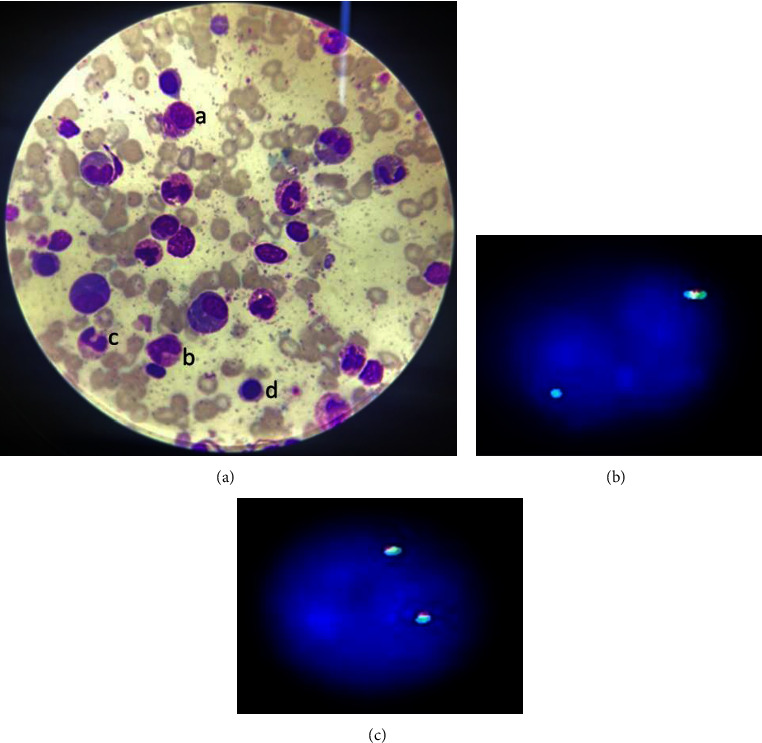
Bone marrow aspirate morphology and FISH result: (a) Bone marrow aspirate showing (i) eosinophilic myelocyte (ii) eosinophilic metamyelocyte (iii) eosinophilic band (iv) normoblast (b) FISH signal pattern at diagnosis indicative of deletion of CHIC2 gene. FISH signal patterns in nuclei having interstitial deletions of the orange probe target (CHIC2) should be observed as one tri-colour fusion and one green/aqua fusion. (c) FISH signal pattern 3 months post imatinib showing no evidence of CHIC2 deletion.

A full blood count (FBC) showed a haemoglobin (Hb) concentration of 11.2 g/dl, total white blood cell (WBC) count of 153 × 10^9^ cells/L (neutrophil count of 112 × 10^9^ cells/L; eosinophil count of 30 × 10^9^ cells/L), and a platelet count of 104 × 10^9^ cells/L (Table 1). A leucoerythroblastic picture with a complete spectrum of myeloid cells was seen on the peripheral film. Bone marrow aspirate revealed a hypercellular marrow with increased granulocytes, 50% of which were comprised of eosinophils at different stages of development. No blasts were noted (Figure 3). RT-PCR for *BCR-ABL* transcripts was negative. FISH analysis was positive for deletion of *CHIC2* in 67% of interphase cells analysed (Figure 3). A diagnosis of chronic eosinophilic leukemia complicated by cardiac failure secondary to valvular heart disease was therefore made.

Before his arrival at our facility, he had received a course of ivermectin that proved ineffective. We initiated treatment with 1 g hydroxyurea twice daily to which the patient showed good haematologic response. After receipt of his FISH analysis, hydroxyurea was replaced with 400 mg daily of imatinib. Repeat FISH analysis 3 months after starting imatinib showed no evidence of *CHIC2* deletion in 100% of the interphase cells analysed (Figure 3). He has not required blood product transfusion since starting treatment and experienced no adverse drug effects of imatinib that required interruption or adjustment of therapeutic dosage. His cardiac failure is being managed with digoxin, diuretics (furosemide and spironolactone), and bisoprolol. Our patient has been unable to undergo a mitral valve replacement due to lack of funds. However, with the above mentioned treatment regimen, he has returned to his normal daily activities and is able to undertake tasks with no functional limitations.

## 3. Discussion

Hypereosinophilia may be reactive, clonal, or idiopathic. Helminthic infection is a well-recognized cause of reactive eosinophilia especially in developing countries. It is worthwhile to note that nonmyeloid malignancies such as T-cell lymphomas and Hodgkin disease can cause nonclonal eosinophilopoiesis due to the production of cytokines such as interleukin-5 which stimulate eosinophil precursor cell proliferation [6]. After exclusion of a reactive cause, investigation for a clonal cause should begin (Table 2).

A peripheral blood film and bone marrow aspirate examination may indicate well-defined WHO myeloid neoplasms such as chronic myeloid leukemia, acute myeloid leukemia, or systemic mastocytosis that may present with eosinophilia. These conditions usually have characteristic genetic markers such as the Philadelphia chromosome [7] or *KIT* gene mutations [8] that must be further investigated.

The Revised 2016 World Health Organization Classification of Eosinophilic Disorders recognizes myeloid/lymphoid neoplasms with eosinophilia and rearrangement of *PDGFRA*, *PDGFRB*, or *FGFR1*, or with *PCM-JAK2* as a distinct clinical entity. Chronic eosinophilic leukemia, not otherwise specified, and idiopathic hypereosinophilic syndrome are diagnoses of exclusion which require the elimination of a known reactive process, genetically defined myeloid malignancy, or a lymphocyte-variant hypereosinophilia (the clonal T-cell population produce a cytokine driven reactive eosinophilia) [9]. Chronic eosinophilic leukemia, not otherwise specified, and idiopathic hypereosinophilic syndrome are similar in that both conditions are associated with persistent hypereosinophilia (absolute eosinophil count >1.5 × 10^9^ cells/L) with evidence of tissue damage. The exact underlying mechanisms of injury have not been well elucidated. It is postulated to be related to direct eosinophil infiltration, fibrosis, or thrombosis ultimately affecting a diverse array of organs including the cranial nerves, oesophagus, lungs, kidneys, skin, and heart [10]. As seen in our patient, there have been infrequent reports of polyarthritis occurring with eosinophilia but without evidence of systemic involvement that is characteristically unresponsive to NSAIDS but ameliorated by corticosteroids [11, 12]. The distinction is made in favour of chronic eosinophilic leukemia, not otherwise specified, when a clonal cytogenetic or molecular genetic abnormality is established, or blast count is ≥ 2% in the peripheral blood or >5% in the bone marrow [2]. In the absence of tissue damage, the term idiopathic hypereosinophilia is preferred.

The *FIP1L1-PDGFRA* fusion gene, a constitutively activated tyrosine kinase, arises as a result of a deletion on chromosome 4q12 [13]. The *CHIC2* gene is located in this region. As such, the *FIP1L1-PDGFRA* fusion gene is often referred to as “CHIC2 deletion” [14]. This is usually detected with FISH but can also be detected by RT-PCR. A significant majority of the patients with this fusion gene are sensitive to tyrosine kinase inhibitors [5] and primary or secondary resistance to imatinib is rare [15]. Discontinuation of therapy may, however, lead to relapse [16].

## 4. Conclusion

In conclusion, physicians should be on high alert when eosinophilia occurs with end-organ damage. The laboratory evaluation for such patients, after elimination of reactive causes, should include screening for the *FIP1L1-PDGFRA* fusion gene, and if found, treatment must be commenced with imatinib. In the absence of a tyrosine kinase inhibitor, hydroxyurea is a viable alternative.

## Figures and Tables

**Table 1:** Series of full blood count results showing response to therapy.

	FBC at presentation	FBC after 2 weeks on hydroxyurea	FBC after 1 week on imatinib	FBC after 1 month on imatinib	Reference range
Hb concentration (g/dL)	11.2	7.9	10.3	10.2	11.0–18.0
WBC count (x 10^9^/L)	153.17	7.05	5.84	6.80	2.5–8.5
Eosinophil count (x 10^9^/L)	30.87	2.56	0.53	0.17	0.04–0.4
Neutrophil count (x 10^9^/L)	112.43	2.43	1.82	3.13	2.0–7.0
Lymphocyte count (x 10^9^/L)	4.60	1.66	3.15	3.00	1.0–3.0
Basophil count (x 10^9^/L)	0.84	0.09	0.04	0.03	0.02–0.2
Monocyte count (x 10^9^/L)	4.29	0.31	0.30	0.47	0.2–1.0
Platelet count (x 10^9^/L)	104	149	271	359	150–450

**Table 2:** Differential diagnoses of hypereosinophilia.

Reactive	Helminthiasis
	T-cell lymphoma
	Lymphocyte-variant hypereosinophilic syndrome
	Hodgkin disease

Clonal	Chronic eosinophilic leukemia
	Chronic myeloid leukemia
	Acute myeloid leukemia
	Systemic mastocytosis

Idiopathic	Idiopathic hypereosinophilic syndrome
	Idiopathic hypereosinophilia

## Data Availability

Deidentified data supporting this case report are available to researchers who meet the criteria for access to confidential data from the corresponding author upon reasonable request.
